# Examining preventive occupational health and safety management in the Swedish welfare sector–questionnaire development, its validity and reliability, and initial findings on employers’ knowledge

**DOI:** 10.1371/journal.pone.0311788

**Published:** 2024-11-14

**Authors:** Magnus Akerstrom, Jonathan Severin, Marta Roczniewska, Ingibjörg H. Jonsdottir, Emina Hadzibajramovic

**Affiliations:** 1 Institute of Stress Medicine, Region Västra Götaland, Gothenburg, Sweden; 2 Institute of Medicine, School of Public Health and Community Medicine, The Sahlgrenska Academy at University of Gothenburg, Gothenburg, Sweden; 3 Department of Learning, Informatics, Management and Ethics, Medical Management Centre, Karolinska Institutet, Procome Research Group, Stockholm, Sweden; 4 Institute of Psychology, SWPS University, Sopot, Poland; Wrocław University of Science and Technology, POLAND

## Abstract

**Introduction:**

A preventive approach to occupational health and safety management (OHSM) can improve working conditions, but more knowledge is needed on how this should be organised in practice. Here, we describe the development, validity and reliability of a questionnaire used to examine employers’ preventive OHSM within the Swedish welfare sector. Furthermore, employers’ knowledge of preventive OHSM was explored using the survey data.

**Material and methods:**

A questionnaire was developed based on interviews with key actors (n = 7), experts (n = 6) and intended respondents (n = 5). Using the final questionnaire, 197 responses were collected from employer (n = 126) and employee representatives (n = 71) and used to assess the validity and reliability of the questionnaire. Qualitative and quantitative analyses of open-ended and multi-choice items were used to assess the response distribution, content validity and interrater reliability (i.e. employer-employee correspondence from 32 matched pairs from the same workplace). Quantitative and qualitative analyses of survey responses from employer representatives were performed to assess their knowledge of preventive OHSM.

**Results:**

The final questionnaire included 91 items covering employers’ working routines, resources and work environment economics. Qualitative analyses indicated a high content validity in the questionnaire, with a satisfactory response distribution and very small proportion of missing data on individual survey items. Overall, the interrater reliability was high (>60%), but the employer representatives generally gave more examples within different areas of the OHSM compared to the employee representatives. Most of the employer representatives (74%, n = 90) assessed that their organisation had sufficient knowledge regarding preventive OHSM but that the knowledge of work environment economics was lower.

**Conclusions:**

By combining work environment research with questionnaire design research, high validity and reliability was achieved for this questionnaire. Furthermore, the employers generally perceived that they have high knowledge of preventive OHSM but that more knowledge is needed on work environment economics.

## Introduction

Working conditions can have both positive and negative effects on the wellbeing of workers [1–3]. In Europe, including Sweden, employers have been obliged by law to provide a safe work environment for more than three decades, where occupational health and safety management (OHSM) must be performed systematically to prevent and manage work-related health problems [4–6]. Nevertheless, work-related mental health problems remain a global challenge, with many workplaces struggling with increasing rates of sickness absence and high employee turnover, particularly in the public sector [1,7,8]. Meanwhile, it is estimated that approximately 200,000 new employees will be required in the Swedish welfare sector (i.e. healthcare, education, social services and public administration) by 2026 to meet the needs of a growing and aging population, worsening the situation even further within this sector [9]. This challenging situation is not unique to Sweden, and the World Health Organization (WHO) estimates that within the healthcare sector alone, there will be a shortfall of 10 million employees globally by 2030 [10]. Thus, attracting and retaining employees within the welfare sector is important, since a shortage of employees will not only affect the health of individual employees, it may also have negative effects on healthcare, including access to services, and the efficacy and quality of care [11–13]. In addition, these health problems result in considerable costs for both employers and society [14].

In Sweden, all employers are obliged by law to systematically assess, improve and monitor employee workload to prevent work-related mental health problems [15]. Since 2015, the regulatory provision also states that preventive OHSM shall be carried out within the organisational and social work environment, with a focus on how work is organised, designed and managed [16], rather than simply strengthening individual employees [17–19].

Despite these regulatory, ethical and operational incentives [20], sickness absence remains higher in the welfare sector compared to other sectors in Sweden [21]. Insufficient integration of the Swedish OHSM regulations in practice has been suggested as one factor contributing to this situation [22,23], especially within organisational and social work environment management, which still focus predominately on OHSM at an individual level [24,25]. Employer knowledge of preventive OHSM is another important factor for successful integration of the OHSM in practice [26]. Thus, increased knowledge is needed on how employers can organise, execute and integrate preventive OHSM in practice to retain and attract employees by providing sustainable working conditions within the welfare sector [16,17,27]. To gain such knowledge, valid and reliable methods are needed for the examination of employers’ preventive OHSM.

The present study was carried out in three parts in order to:

1. describe the development of a questionnaire used to examine employers’ preventive OHSM within the Swedish welfare sector,
2. perform data collection and empirical evaluation of the questionnaire items, and
3. explore employers’ knowledge of preventive OHSM.

The scope of this study included employers’ preventive OHSM measures in response to the Swedish provision of systematic work environment management [4] with a focus on the initiation, design and implementation of measures within preventive OHSM, along with the associated work environment economics.

## Part 1: Development of a questionnaire to examine employers’ preventive OHSM within the Swedish welfare sector

In the initial phase, the research group focused on 1) identifying the key concepts within the scope of the study and 2) constructing the questionnaire by formulating items and response options. To improve the validity and reliability of the questionnaire, tests were conducted at various stages of the process.

### Conceptualisation and identification of key concepts

When reviewing the literature, no validated questionnaire that covered the scope of this study was found. Previously, knowledge on employers preventive OHSM have mainly been derived from qualitative studies of single or a limited number of cases without the use of quantitative questionnaires [22,28]. Among the few studies that had used quantitative questionnaires for investigating the employers OHSM, sufficient information on the validation process was lacking and the questionnaires focused largely on the presence of specific routines instead of the organisation of the OHSM and its performance in practice [22,28]. To develop a questionnaire that covered the scope of this study, interviews with seven stakeholders within the welfare sector (managers [n = 3], human resources [HR, n = 2], a client manager [responsible for supporting their clients in contractual and strategical decisions within the OHSM] and an organisational psychologist within the occupational health [OH] services) were performed to identify key concepts within the scope of the study. These stakeholders were selected to represent the different actors within the OHSM (i.e., managers, employees, support functions and the OH services) and all had a thorough experience within their respective role. Interviews were performed until no new information was derived and the collected data was saturated. Based on the interviews, the research group also discussed which aspects could be measured with a questionnaire and which aspects could best be handled with other OHSM tools. The interviews were conducted digitally by project members with extensive knowledge of OHSM and interview techniques. Each interview was conducted using a semi-structured approach with questions concerning the organisation and execution of preventive OHSM (S1 File). The interviews lasted approximately 60 minutes and were analysed using deductive qualitative analysis [29] based on the themes participation, work environment policy and routines, allocation of tasks, knowledge, risk assessment, measures, and follow-up within the provision of work environment management in Sweden [4].

The qualitative analysis of the interview data resulted in six key concepts: organisational prerequisites (e.g. challenges with the work environment and use of occupational health service, sector or industry), cooperation (e.g. between managers and HR or with OH services), knowledge (e.g. about work environment management or workplace interventions), routines (e.g. organisation of work environment management, measurable goals and evaluation of interventions), incentives (e.g. motives for implementation and investment in workplace interventions) and performance (e.g. prevention of illness, implementation of interventions and estimation of costs).

### Formulating and testing questionnaire items

In the next phase, the project team operationalised the identified key concepts into specific questionnaire items. The items were formulated to reflect the content of the six key concepts above and their connected sub-themes identified in the qualitative analysis of the interviews with the stakeholders. The items were initially formulated by one of the authors (JS) which also performed most of the interviews, using his experiences and knowledge of how the language and wording is interpreted by the intended respondents. These items were then reviewed and discussed in the research group until consensus was reached. This resulted in a total of 86 items covering the six key concepts. To get a full understanding, these items included contextual (i.e. items connected to the size and type of operations differentiating one workplace from another), structural (i.e. items related to how OHSM has been organised on a structural level and access to resources), processual (i.e. items related to the implementation of measures within OHSM) and outcome items (i.e. items related to the outcomes of OHSM). These items covered the practical design of preventive OHSM and items instructing the respondents to make expert assessments on the performance of their OHSM based on their expertise within the area and the organisation. Based on the interviews, multiple-choice responses were used to describe how the OHSM was designed in practice. For the expert assessments, five Likert-scale response alternatives ranging from “to a very low degree” to “to a very high degree” were considered to be the most suitable response alternatives as the respondents were able to elaborate on their responses rather than giving a yes/no response. Moreover, five open-ended control items were added as an additional posterior check of the questionnaires content validity, that is, whether the respondents gave examples of preventive and organisational-level measures within the scope of the study in accordance with the identified six key concepts. In the open-ended items, respondents were asked to provide brief examples of preventive and organisational-level work environment measures that have been implemented or are planned in their organisation, the estimated total cost of work environment measures and what they thought was needed to improve their use of work environment economics and preventive OHSM. Thus, a total of 91 items were included in the first draft of the questionnaire, which were then evaluated in an expert review.

An expert review of the formulated items was carried out by a total of six individuals representing researchers (n = 2), managers (n = 1), the union (n = 1), HR (n = 1) and the OH services (n = 1). These experts were selected to represent the different actors within the OHSM (i.e., managers, employees, support functions and the OH services) as well as researchers specialised within the field. All experts had a thorough experience within their respective role and the expert review was performed until no new information was derived and the collected data was saturated. The focus of this review was on content validity (e.g. whether the items seemed relevant and properly covered the scope of the study and the six identified key concepts, and whether something is missing or should be removed), response psychology (e.g. whether respondents understand the questions, can make judgements about the information and formulate responses) and the cognitive and organisational response process in surveys targeting organisations (e.g. how to retrieve relevant information that is asked for in the items). For the latter, the hybrid response process model [30] was used, which combines the four-step model developed by Tourangeau [31] (covering comprehension, retrieval, judgement and response) with additional organisational factors concerning the organisations’ selection of respondent, assessment of priorities and comprehension of the data request. The respondents were also asked to provide written feedback.

The expert review showed an overall high content validity and useability with only minor revisions of single items, where response alternatives were suggested, and the questionnaire was revised accordingly.

Lastly, five potential responders (managers [n = 2], HR [n = 2] and OH services [n = 1]) within the welfare sector pilot tested the questionnaire and provided their written feedback. The pilot testing showed an overall high content validity and functionality, and only minor revisions of single items and response alternatives were suggested. The questionnaire was revised to improve both the functionalities of the electronic survey and the content validity, resulting in a final version of the questionnaire (S2 File), which was used in the data collection described below.

## Part 2: Data collection and empirical evaluation of the items

### Data collection and study population

To collect data, a link to the electronical survey was distributed through e-mails directly to all Swedish municipalities, regions, and privately-owned businesses that operate within the public funded welfare sector (retrieved from public records and registers from the Swedish Association of Local Authorities and Regions, The Health and Social Care Inspectorate and The Swedish National Agency for Education) and advertised through social media (Facebook and LinkedIn) on the research group’s external webpage, via unions and other professional networks, and through external collaborations. This study was approved by the Swedish Ethical Review Authority (Dnr 2023-00974-01). The organisations participated after providing written informed consent in the electronical survey and nominated one or more employer and employee representatives (used to assess the interrater reliability; see part 2 below) to answer the survey depending on the organisation of their OHSM. The representatives with the most thorough knowledge of their organisations’ preventive OHSM were asked to respond to the survey on behalf of the organisation. The survey was open for two months, (17 April through 17 June 2023) and used a quota sampling strategy with the goal to reach about 150 responses within the different sectors (public, municipality and region). This goal was based on previous studies and experiences with the aim of being able to perform sub-analyses on the different sectors.

In total, 197 responses were collected in the survey, where 126 came from employer representatives and the remainder from employee representatives. In total, 10 responses (5%) were excluded due to missing informed consent (n = 4, 2%), participating workplace not belonging to the welfare sector (n = 4, 2%) and responding to <50% of the items within the survey (n = 2, 1%), resulting in responses from 122 employer and 65 employee representatives. Among these, 32 pairs with responses from employer and employee representatives from the same workplaces were identified. Responses came from workplaces located in 20 of 21 regions in Sweden, mainly working with public administration, education and healthcare. In the private sector, the employer representatives were most often managers (68%), and in municipalities and regions, members of HR were mainly assigned to be employer representatives (89% and 78%, respectively; p<0.001 for differences between sectors). Employer representatives were mainly safety delegates. Almost all the participating workplaces expressed challenges related to employee workload and fewer reported challenges related to the social and physical work environment. Descriptive background results for employer and employee representatives can be seen in Table 1.

**Table 1:** Descriptive background results for employer and employee representatives.

	Employer representatives					Employee representatives				
	Total	Private sector	Municipal sector	Regional sector	p-value for difference between sectors^a^	Total	Private sector	Municipal sector	Regional sector	p-value for difference between sectors^a^
**Workplaces, n (%)**	122 (100)	25 (20)	74 (61)	23 (19)		65 (100)	11 (17)	38 (58)	16 (25)	
**Industry, n (%)**					<0.001					0.06
Culture, entertainment and leisure	2 (2)	0 (0)	0 (0)	2 (9)		0 (0)	0 (0)	0 (0)	0 (0)	
Labour-intensive service (rental, property services, support services and other services)	3 (2)	0 (0)	2 (3)	1 (4)		0 (0)	0 (0)	0 (0)	0 (0)	
Knowledge-intensive service (law, science, technology, finance and real estate operations)	2 (2)	0 (0)	1 (1)	1 (4)		0 (0)	0 (0)	0 (0)	0 (0)	
Public administration	61 (50)	0 (0)	57 (77)	4 (17)		23 (40)	1 (9)	20 (53)	5 (31)	
Education	12 (10)	10 (40)	2 (3)	0 (0)		8 (12)	4 (36)	4 (11)	0 (0)	
Health and care; social services	41 (34)	15 (60)	11 (15)	15 (65)		27 (42)	4 (36)	13 (34)	10 (63)	
Other^b^	1 (1)	0 (0)	1 (1)	0 (0)		4 (6)	2 (18)	1 (3)	1 (6)	
**Role answering the survey, n (%)**					<0.001					0.4
Work environment coordinator	9 (7)	4 (16)	4 (5)	1 (4)		0 (0)	0 (0)	0 (0)	0 (0)	
HR	88 (72)	4 (16)	66 (89)	18 (78)		0 (0)	0 (0)	0 (0)	0 (0)	
First-line manager	16 (13)	12 (48)	1 (1)	3 (13)		0 (0)	0 (0)	0 (0)	0 (0)	
Higher manager	9 (7)	5 (20)	3 (4)	1 (4)		0 (0)	0 (0)	0 (0)	0 (0)	
Safety delegate	0 (0)	0 (0)	0 (0)	0 (0)		49 (75)	8 (73)	27 (71)	14 (88)	
Union representative	0 (0)	0 (0)	0 (0)	0 (0)		16 (25)	3 (27)	11 (29)	2 (12)	
**Workplace size, n (%)**					<0.001					<0.001
Small: up to 19 employees	10 (8)	8 (32)	2 (3)	0 (0)		4 (7)	1 (11)	2 (6)	1 (7)	
Medium/small: 20–49 employees	13 (11)	12 (48)	0(0)	1 (4)		6 (10)	3 (33)	3 (9)	0 (0)	
Medium: 50–249 employees	11 (9)	4 (16)	3 (4)	4 (17)		8 (14)	4 (44)	2 (6)	2 (14)	
Large: 250 or more employees	86 (72)	1 (4)	67 (93)	18 (78)		40 (69)	1 (11)	28 (80)	11 (79)	
**Workplace challenges, n (%)**										
Workload^c^	113 (93)	21 (84)	70 (95)	22 (96)	0.2	60 (92)	10 (91)	35 (92)	15 (94)	1.0
Social work environment^d^	77 (63)	10 (40)	54 (73)	13 (57)	0.01	39 (60)	4 (36)	28 (74)	7 (44)	0.03
Physical work environment^e^	43 (35)	4 (16)	31 (42)	8 (35)	0.06	25 (38)	3 (27)	16 (42)	6 (38)	0.7

^a^Assessed using chi2-test (for binomial variables) or Kruskal-Wallis test (for non-bionomal variables)

^b^Construction industry for employer representatives and transport, manufacturing, trade and hotel and restaurant operations for employee representatives

^C^An imbalance between job demands and job resources

^d^Threats and violence, harassment and bullying and conflicts

^e^Physical strain, noise, air, and chemical exposure, and accidents and safety risks.

The validity and reliability were then assessed in three steps by 1) analysing the response distribution on questionnaire items, 2) evaluating the open-ended control question and 3) evaluating the interrater reliability.

### Response distribution on questionnaire items

All responses were systematically reviewed to ensure that only valid responses were collected, and the degree of missing data was observed. Overall, all response alternatives showed a satisfactory distribution. The proportion of responders using response alternatives corresponding to “I don’t know” was low on most items (generally below 5%), except for items concerning work environment economics within the organisations’ OHSM (about 20%). The measurement error due to invalid responses and partial missing data (i.e., when a respondent chose not to respond to single questions or sections within the survey) were low (generally below 5%). For one item, “Which industry best describes your organization?”, a less satisfactory response distribution and a larger proportion of non-valid responses were seen.

### Evaluation of open-ended control questions

The content validity was assessed using the employer representatives’ responses on three open-ended items concerning 1) examples of implemented preventive measures within the organisation’s OHSM, 2) examples of implemented organisational-level measures within OHSM and 3) costs included when estimating the total cost of work environment measures. The open-ended items were analysed using inductive content analysis according to Elo and Kyngäs [29]. An example of the inductive qualitative analysis process is shown in (S3 File).

Most of the respondents provided examples of implemented preventive and organizational-level measures within their organisation’s OHSM (95% and 83% response rate, respectively). The qualitative analyses of the first two open-ended items resulted in five sub-themes: *Development of the systematic preventive and promotional work environment management*, *Gaining knowledge of working conditions*, *Workplace interventions*, *Increased access to support and HR practices* (Tables 3 and 4), which corresponded well to the key concepts identified in the development of the questionnaire (Part 1). Within all sub-themes, identified sub-categories could be divided into structural measures or dialogue and individual level measures. Although they covered the same sub-themes, the proportion of structural measures was higher for the examples of organisational-level measures (63%) compared to examples of preventive/promotive measures (52%), which was expected. The largest difference was seen for the sub-theme, Workplace interventions where 57% of the examples of organisational level measures were structural compared to 30% of the examples of preventive measures (Tables 2 and 3).

**Table 2:** Result of the qualitative analysis on examples of implemented preventive measures.

Theme	Sub-theme	Category	Sub-category
Preventive measures within systematic work environment management (n = 538)	Development of systematic preventive work environment management (n = 100)	Structural measures (n = 83)	Work routines (n = 63)
			Work tools (n = 13)
			Digitalisation (n = 7)
		Dialogue and individual measures (n = 17)	Participation (n = 8)
			Life-style changes (n = 9)
	Gaining knowledge of working conditions (n = 120)	Structural measures (n = 96)	Investigations and analysis (n = 41)
			Surveys (n = 40)
			Safety inspections (n = 15)
		Dialogue and individual measures (n = 24)	Workplace dialogues (n = 14)
			Employee participation (n = 10)
	Workplace interventions (n = 236)	Structural measures (n = 70)	Work practices (n = 24)
			Working time arrangements (n = 9)
			Recovery (n = 6)
			Physical work environment (n = 31)
		Dialogue and individual measures (n = 166)	Group development (n = 32)
			Health-promotion interventions (n = 23)
			Education (n = 108)
			Rehabilitation (n = 3)
	Increased access to support (n = 39)	Structural measures (10)	Internal support (n = 2)
			External support (n = 8)
		Dialogue and individual measures (n = 29)	Support to managers (n = 17)
			Support to employees (n = 12)
	HR practices (n = 43)	Structural measures (n = 23)	Benefit programmes (n = 23)
		Dialogue and individual measures (n = 20)	Physical activities (n = 20)

**Table 3:** Result of the qualitative analysis on examples of implemented organizational level measures.

Theme	Sub-theme	Category	Sub-category
Organisational-level measures within systematic work environment management (n = 398)	Development of systematic preventive work environment management (n = 91)	Structural measures (n = 66)	Work routines (n = 53)
			Work tools (n = 13)
		Dialogue and individual measures (n = 25)	Participation (n = 22)
			Life-style changes (n = 3)
	Gaining knowledge of working conditions (n = 86)	Structural measures (n = 66)	Investigations and analysis (n = 31)
			Surveys (n = 17)
			Safety inspections (n = 18)
		Dialogue and individual measures (n = 20)	Workplace dialogues (n = 11)
			Employee participation (n = 9)
	Workplace interventions (n = 169)	Structural measures (n = 96)	Work practices (n = 28)
			Organisation of work (n = 29)
			Operations design (n = 37)
			Physical work environment (n = 2)
		Dialogue and individual measures (n = 73)	Group development (n = 9)
			Education (n = 62)
			Rehabilitation (n = 2)
	Increased access to support (n = 38)	Structural measures (9)	Internal support (n = 4)
			External support (n = 5)
		Dialogue and individual measures (n = 29)	Support to managers (n = 5)
	HR practices (n = 14)	Structural measures (n = 12)	Health-promotion practices (n = 8)
			Benefit programs (n = 3)
		Dialogue and individual measures (n = 2)	Physical activities (n = 2)

Only about half of the respondents (52%) provided information on the costs included when estimating the total cost of work environment measures. Among these answers, about 20% could not provide any relevant information (e.g. “I don’t know”, “not relevant”, etc.). The quantitative analysis of the remaining responses identified four sub-themes that corresponded to the intended key concept: *Purchase cost*, *Capacity building costs*, *Operational costs* and *Costs for internal and external support* (Table 4).

**Table 4:** Results of the qualitative analysis of costs included when estimating the total cost of work environment measures.

Theme	Sub-theme	Category
Costs included when estimating the total cost of work environment measures (n = 142)	Purchase costs (n = 50)	Interventional costs (n = 17)
		Material costs (n = 12)
		Rental and venue costs (n = 10)
		Costs for investments (n = 5)
		Costs for food and drinks (n = 4)
		IT systems-related costs(n = 2)
	Capacity building costs (n = 16)	Education costs (n = 11)
		Costs for invited lecturers (n = 3)
		Training costs (n = 2)
	Operational costs (n = 54)	Costs for productivity loss (n = 7)
		Costs for working time (n = 20)
		Costs for temporarily employed staff (n = 10)
		Costs for staff salaries (n = 10)
		Costs for sickness absence (n = 5)
		Travel costs (n = 2)
	Costs for internal and external support (n = 22)	Costs for external consultants (n = 13)
		Costs for occupational health services (n = 5)
		Costs for internal support (n = 4)

Thus, the results showed an overall high content validity for preventive and organisational-level OHSM but a somewhat lower content validity for the work environment economics.

### Interrater reliability

The interrater reliability was assessed by investigating the employer-employee correspondence for a sub-set of 32 pairs (responses from employer and employee representatives from the same workplaces). These include pairs from the private sector (n = 7), municipal sector (n = 18) and regional (n = 7) sector. The employer-employee correspondence was assessed using deductive qualitative content analyses according to Elo and Kyngäs [29] based on the similarities between responses (yes/no) on the 86 items. These assessments were performed separately by two of the authors (MA and JS). The assessments were then compared, and potential differences were discussed upon agreement.

There was a high employer-employee correspondence (66%– 100%) for all contextual questionnaire items. For the structural items, a high correspondence (>60%) was seen for most items, but the employee representatives reported “do not know” to a higher degree on items related to collaboration with OH services and reported somewhat lower knowledge and lower access to working methods and routines, resulting in a slightly lower correspondence (53% and 47%, respectively). For the processual items, the correspondence was high overall (mostly >60%), but the employer representatives provided a wider range of examples compared to the employee representatives. This was also seen when the employer-employee correspondence was assessed for the open-ended items, which had a correspondence of 83% for the examples of implemented preventive measures. However, the employer representatives again showed a tendency to provide more examples within different areas of OHSM compared to the employee representatives. For the outcome variables in the survey, employers and employee representatives shows a relatively high level of agreement (69% and 63%, respectively) regarding the proportion of implemented preventive and organisational-level measures. For the preventive measures, the employer representatives had a somewhat higher assessment compared to the employee representatives in case of disagreement, but no systematic difference was seen in case of disagreement for the proportion of implemented organisational-level measures. Lastly, the employer representatives assessed that their preventive OHSM prevented health problems to a higher degree and promoted health compared to the employee representatives, resulting in employer-employee correspondences of 47% and 50%, respectively.

## Part 3: Exploration of employer’s knowledge of preventive OHSM

The employers’ practical knowledge of preventive OHSM was examined by qualitative and quantitative analyses of the results from Part 2 (see above) and the employer representatives’ survey responses on items connected to employers’ knowledge of preventive OHSM.

### Method

To examine employer knowledge of preventive OHSM, deductive qualitative analyses were performed according to Elo and Kyngäs [29]. These analyses were based on the provision of work environment management in Sweden [4] and examined the employer representatives’ responses to the open-ended items concerning examples of implemented preventive and organisational-level measures and the costs included when estimating the total cost of work environment measures (Tables 2–4). In addition, the employer representatives’ survey responses from two items on the questionnaire were used: “In my organisation, we have sufficient knowledge to select relevant measures in preventive/promotive work environment management”, and the open-ended item, “What do you think would make your preventive/promotive work environment management more successful?” These survey responses were analysed quantitatively, and potential differences between sectors (private, regional and municipal) were investigated using chi2-test (for binomial variables) or Kruskal-Wallis test (for non-binomial variables).

### Results

The qualitative analyses on examples of implemented preventive and organisational-level measures showed that the employer representatives tended to operationalise the concepts of preventive measures and organisational-level measures within the organisation’s OHSM. In addition, there was a difference observed between the provided examples for these two key concepts, with a larger proportion of structural measures for organisational-level measures compared to preventive measures (Tables 2 and 3). Meanwhile, the qualitative analyses revealed limited knowledge of work environment economics within the organisation’s OHSM (Table 4).

The employer’s knowledge could also be assessed using specific items in the survey. Most of the employer representatives (74%, n = 90) assessed that their organisation had the necessary knowledge to design efficient preventive measures within preventive OHSM. There was no difference between the private, municipal and regional sectors (72%, n = 18; 72%, n = 53 and 86%; n = 19, p = 0.4, respectively). In addition, the need for increased knowledge was mentioned in less than 20% of responses to the open-ended item “What do you think would make your preventive/promotive work environment management more successful?” (14 out of 77 responses).

## Discussion

This study describes the development of a questionnaire to be used to examine employers’ preventive OHSM within the Swedish welfare sector. The validity and reliability of the questionnaire was also assessed. Results show that a questionnaire can be an effective way to collect data required for research and the evaluation of the employers’ preventive OHSM. By combining work environment research and questionnaire design research, high validity and reliability was achieved. In addition, employers’ practical knowledge concerning preventive OHSM was investigated. Overall, the results showed that employers are perceived to a have a high degree of knowledge of the key concepts within preventive OHSM, though more knowledge is needed on work environment economics.

Questionnaires can be an effective way to collect the information or data needed for research and evaluation. However, ensuring that a well-developed questionnaire is used is a crucial step to avoid measurement errors and facilitate the accurate interpretation of results [32–34]. Therefore, when publishing findings based on survey studies, it is important to describe the development process of the questionnaires in sufficient detail and with sufficient rigor in order to enable practitioners to interpret results obtained from questionnaires and make informed decisions about whether to implement the findings or not [33]. There are several challenges associated with developing a questionnaire with high validity, reliability and usability in connection with the cognitive response process [31]. One challenge associated with operationalising key concepts in a study is that it requires a shared understanding of how the language and wording is interpreted by the researchers and the intended respondents [33]. To minimise the risk of misinterpretation, representatives from the intended respondents were included in the development process, not just during the pilot testing of the final questionnaire. This resulted in the inclusion of work environment economics in addition to more traditional aspects of OHSM, which is an important but often forgotten aspect of preventive OHSM in practice [35].

To improve the validity and reliability even further, the questionnaire was also reviewed by experts with a focus on the content validity, response psychology and cognitive and organisational response process in surveys [32]. Since the questionnaires targeted organisations rather than individuals, special attention had to be given during the development process to organisational challenges linked to internal processes within the organization, such as selection of respondents, assessment of priorities and comprehension of the data request [30].

To ensure sufficient validity and reliability in the final questionnaire, the response distribution for items, content validity and interrater reliability were assessed. The response distribution was found to be satisfactory. When investigating the content validity, five sub-themes of implemented preventive and organisational measures were found, which were well in line with the key concepts of the study and Swedish regulations [4,15], indicating a high content validity. Thus, practitioners or researchers with an interest in OHSM in the Swedish welfare sector may use this questionnaire in similar organisations to gain increased knowledge of preventive OHSM. In addition, other practitioners or researchers may use our items as inspiration when developing a similar questionnaire for other contexts.

The identified sub-themes in preventive and organisational measures covered both the development of overall practices and routines within preventive OHSM and specific aspects, such as increased access to support and HR, measures aimed at increasing knowledge of working conditions and the implementation of preventive measures. Although access to efficient practices and routines within OHSM is important [36], concrete measures need to be implemented to create sustainable working conditions that attract and retain employees within the welfare sector [16]. Consequently, our findings may indicate that many employers still focus on identifying efficient practices and routines rather than improving specific adverse working conditions, even though most workplaces described their workload as their main challenge. The need for increased knowledge of how OHSM can be organised and executed in practice has also been recognised in previous studies [16,17,27]. If implemented successfully, measures aiming to develop practices and routines within preventive OHSM may, in the long run, result in improved workings conditions for employees by increasing the opportunity to identify preventive measures in the work environment. However, these measures should preferably be combined with measures targeting the main challenges in the work environment [37]. In addition, whether the observed measures were designed and implemented successfully in practice could not be investigated in the current study. It should also be noted that the respondents only provided examples of implemented measures and not a complete summary of all implemented measures within their organisation.

Within each of the five sub-themes, structural measures, individual measures and dialogue were present. Looking at the proportion of measures on a structural level could indicate that individual and group-level measures still constitute a large part of measures that are implemented in practice. However, this conclusion should be interpreted with caution, as the analyses are based on examples and not a complete list of implemented measures. In addition, improving adverse working conditions often require measures on multiple levels to reach maximum impact [3,38].

The content validity concerning the economic considerations was somewhat lower, mainly because of the limited response rate. Only about 50% of respondents provided information on the economic considerations they weighed when designing preventive/promotional measures within their organisation’s OHSM, and one out of five of these did not provide any relevant information, underscoring the need to create a better balance between economic considerations and health economic assessments within the organisation’s OHSM [35].

There was an overall high interrater reliability, measured as the correspondence between the employer and employee representatives’ responses. However, employee representatives were found to have a more limited overview of practices and activities within the organisation’s OHSM compared to the employer representatives. This may indicate a challenge in finding suitable employee representatives or may indicate the weak integration of Swedish OHSM regulations in practice, which has been suggested in previous studies [22,23].

The employer’s knowledge of preventive OHSM was investigated using the data collected in the final questionnaire. Our results indicate that employers report an overall high level of knowledge of preventive OHSM. Furthermore, qualitative analyses indicated that they operationalised the content of Swedish regulations into interventions in their own organisation and were able to provide valid and reliable responses to the survey items concerning the initiation, design and implementation of preventive and promotive OHSM. In line with previously identified supporting factors for organisational-level interventions (see von Thiele Schwartz et al. [37] for an overview), sufficient knowledge of preventive OHSM is an important prerequisite. Similarly, Wikström et al. [26] found that sufficient knowledge of preventive and promotive OHSM is a supportive factor in the pre-intervention phase of an organisational-level intervention within the public sector in Sweden.

However, possessing such knowledge may not be enough to ensure the successful implementation of OSHM. Knowledge of implementation strategies and change management is also needed for maximum effect [26,37]. In our data, three out of four respondents assessed that their organisation possessed sufficient knowledge to design efficient intervention measures. However, when reviewing the provided examples of implemented measures, most measures focused on finding efficient practices and routines in the preventive OHSM. Thus, more knowledge is needed to determine how this self-assessed knowledge can be converted into practice [17,39].

Regarding knowledge of work environment economics, the employers’ knowledge was lower compared to other areas within preventive OHSM, and most of the respondents did not provide examples of their organisation’ practices within this area. Work environment economics (e.g. effort-gain analysis) are an important but often overlooked part of the design of organisational-level interventions [37]. There is a need to quantify the economic costs of a poor work environment and the economic benefits of a good work environment to a larger degree in order to illustrate the magnitude of the economic loss that comes with a health-hazardous work environment, and vice-versa [35]. Such knowledge may then increase the incentives for employers to invest in preventive OHSM.

### Strengths and limitations

A strength of this study is the close collaboration with intended respondents during all parts of the development process and the combination of work environment research and questionnaire design research, which was used to improve the validity and reliability of the survey. Even though the quotas could not be filled for the private and regional sector, the distribution of responses covered most regions of Sweden and different parts of the welfare sector. One reason the quotas were not filled is the current strained economic situation within the Swedish welfare sector, resulting in limited opportunities for organisations to prioritise non-core activities. Another limitation is the limited numbers of paired responses from employer and employee representatives, which was not possible to increase since the employers themselves nominated respondents and no reminders could be sent. Lastly, we found support for the claims in the questionnaires and content validity and reliability, but other types of validity should be examined in future research. For instance, to establish the predictive validity of the instrument, studies could test whether scores obtained with this instrument predict relevant workplace outcomes that are likely to be linked to preventive OSHM, such as number of accidents or sick leave statistics.

## Conclusions

Questionnaires can be an effective way to collect the information or data needed for research and evaluation. However, in order to ensure high data quality and the utilisation of research outputs, a thorough development process is needed. By combining work environment research with questionnaire design research, high validity and reliability were achieved for this questionnaire. The results indicate that employers report an overall high knowledge of preventive OHSM. Furthermore, qualitative analyses indicated that employers operationalised the content of Swedish regulations into interventions that were implemented in their organisations. Despite this, a high proportion of the examples of implemented measures concerned the development of practices instead of measures targeting current challenges with an adverse workload. Lastly, the knowledge of work environment economics within OHSM was generally low. In order to fully realise the potential of preventive OHSM, increased knowledge of work environment economics is needed.

## Supporting information

S1 File Interview guide. (PDF)

S2 File The questionnaire. (PDF)

S3 File Examples of the inductive qualitative analysis process. (PDF)

S4 File Dataset. (PDF)
