# Supplementary material for: Examining preventive occupational health and safety management in the Swedish welfare sector–questionnaire development, its validity and reliability, and initial findings on employers’ knowledge
Source: PLoS One. 2024 Nov 14;19(11):e0311788. doi: 10.1371/journal.pone.0311788 (PMC11563452; doi:10.1371/journal.pone.0311788)
Supplement: S1 File — (PDF) [file pone.0311788.s001.pdf]

S1 Interview guide for stakeholder interviews

Experience

1. Do you have any experience with preventive measures in the past year? a. Experience with organisational measures? For example, lecture series, reorganisation?

Decisions

2. What have decisions about preventive measures been based on? a. What type of facts or data have been used? For example, surveys, sickness rates, conversations, evidence. b. Other reasons? For example, legal, economic, moral, guidelines. c. What has been important to know when making decisions?

Initiation

3. How has the initiation process been carried out? a. How was the measure determined? b. Who has been involved in the decision? Manager, HR, occupational health service, employees, other support. c. Who has the decision-making authority in case of disagreement about the choice of measure? d. How has the collaboration worked among those involved? e. How does the dialogue take place? For example, meetings, conversations, informal talks.

Implementation

4. How has the implementation been organised? a. Who has been involved in the execution? b. How has follow-up been conducted? c. Experiences when it has gone well or poorly? d. Any obstacles to decisions, initiation, implementation or follow-up?

Work environment economics

5. To what extent have health economic arguments been considered? a. How important has this been? For example, return on investment, profitability and costs. b. How has information

regarding this been gathered? c. What costs have been associated with the measures? d. How have costs been tracked?
