# Supplementary material for: Examining preventive occupational health and safety management in the Swedish welfare sector–questionnaire development, its validity and reliability, and initial findings on employers’ knowledge
Source: PLoS One. 2024 Nov 14;19(11):e0311788. doi: 10.1371/journal.pone.0311788 (PMC11563452; doi:10.1371/journal.pone.0311788)
Supplement: S2 File — (PDF) [file pone.0311788.s002.pdf]

VÄSTRA GÖTALANDSREGIONEN

Survey regarding practices in the preventive and promotive work environment management

Please read through this text before answering the survey.

This survey is aimed at organizations both in the private and public sectors and takes approximately 20 minutes to complete. The survey is intended to be answered by two individuals: firstly, the employer representative who has the best knowledge of how the work environment management is organized, planned, and carried out (e.g., HR, manager, environmental coordinator, or similar); secondly, an employee representative (e.g., safety delegate or union representative).

The questions in the survey should be answered based on the level and scope at which the work environment management is conducted. In a larger organization, this would refer to your department, unit, section, or equivalent.

The purpose of the survey is to investigate how employers proceed when they wish to implement preventive and/or promotive work environment inventions as part of their systematic work environment management, and to identify enabling and hindering factors. The goal of this knowledge is to help employers improve their work environment management, which ultimately can contribute to reducing work-related health problems. We hope that you will contribute by responding to this survey.

The following terms are used in the survey:

Preventive work environment measures refer to all measures aimed at reducing the risk of employees suffering from health problems and/or accidents at work.

Promotive work environment measures refer to measures aimed at maintaining and improving employees' health.

Organizational-level work environment measures refer to measures aimed at influencing how the organization is managed and/or how work and access to resources are organized. These measures can be both preventive and/or promotive.

Examples of preventive and/or promotive work environment measures can include knowledge-enhancing activities, changes in routines and work methods, procurement of aids, changes in the physical work environment, measures aimed at influencing behaviors and community, as well as strengthening well-being, increasing empowerment, or the sense of coherence, among others.

Work environment measures aimed at addressing the consequences of work-related health problems that has already occurred (so-called ***rehabilitative measures) are not covered by this survey***.

1. **Do you approve that your answers can be used in research according to the attached information (see link below)?**

Read more here: Survey information letter

☐ Yes

☐ No

2. The questions in the survey should be answered based on the organization and level at which the work environment management is conducted. In a larger organization, this would refer to your department, unit, section, or equivalent.

Which organization and, if applicable, workplace does your answers pertain to?

Organization (company, region, municipality, etc.):

____

Workplace (section, department, administration, etc.):

____

3. Based on which role are you answering this survey?

- ☐ Safety delegate
- ☐ Union representative
- ☐ Work environment coordinator or similar
- ☐ HR
- ☐ Manager with employee responsibility
- ☐ Higher manage
- ☐ Another role (free text)

Systematic work environment management

4. Which option best corresponds to how you carry out your systematic work environment management?

- ☐ Manager with employee responsibility independently carries out the systematic work environment management without support
- ☐ Manager with employee responsibility independently carries out the systematic work environment management with support from collectively developed routines and work methods
- ☐ Manager with employee responsibility carries out the systematic work environment management with support from internal resources (e.g., HR, work environment coordinator, safety delegate)
- ☐ Manager with employee responsibility carries out the systematic work environment management with support from internal and external resources (e.g., HR, work environment coordinator, safety delegate, occupational health service, external consultants)
- ☐ Manager with employee responsibility has delegated the responsibility for carrying out tasks related to the systematic work environment management to another function

5. Are there measurable goals related to work environment that are regularly monitored in your organization?

- ☐ Yes
- ☐ No
- ☐ Partially
- ☐ Do not know

6. Are any of the set goals related to the organizational and social work environment? (Only answered if yes/partially to question 5)

- ☐ Yes
- ☐ No
- ☐ Partially
- ☐ Do not know

Preventive and promotive work environment management

Preventive/promotive work environment measures refer to all measures aimed at reducing the risk of employees suffering from health problems and/or accidents at work, or at maintaining and improving employees' health.

7. Approximately what proportion of all work environment measures conducted in your organization are preventive/promotive?

<25%	26–50%	51–75%	76–100%	Do not know
☐	☐	☐	☐	☐

8. Which option best describes the extent to which preventive/promotive work environment measures have been implemented in your organization <u>over the past three years</u>?

☐ We have not implemented any preventive/promotive work environment measures in the past three years

☐ We have implemented occasional preventive/promotive work environment measures in the past three years

☐ We have implemented several preventive/promotive work environment measures in the past three years

☐ We have been engaged in continuous improvement with preventive/promotive work environment measures in the past three years

9. Provide brief examples of preventive/promotive work environment measures that <u>have been implemented</u> or <u>are planned</u> in your organization.

____ (free text, 400 characters)

Work environment measures at organizational level

Organizational-level work environment measures refer to measures aimed at influencing how the organization is managed and/or how work and access to resources are organized. These measures can be both preventive and/or promotive.

10. Approximately what proportion of all work environment measures conducted in your organization are at organizational level?

<25%	26–50%	51–75%	76–100%	Do not know
☐	☐	☐	☐	☐

11. Which option best describes the extent to which organizational work environment measures have been implemented in your organization <u>over the past three years</u>?

- ☐ We have not implemented any organizational work environment measures in the past three years
- ☐ We have implemented occasional organizational work environment measures in the past three years
- ☐ We have implemented several organizational work environment measures in the past three years
- ☐ We have been engaged in continuous improvement with organizational work environment measures in the past three years

12. Provide brief examples of organizational work environment measures that <u>have been implemented</u> or <u>are planned</u> in your organization.

____ (free text, 400 characters)

Planning of work environment measures and approaches

These questions focus on how you usually proceed when making decisions about preventive/promotive work environment measures.

13. To what extent are the following functions involved in <u>identifying the need</u> for preventive/promotive work environment measures?

	Very low extent	Low extent	Neither low nor high extent	High extent	Very high extent	Function does not exist
a) Top management	☐	☐	☐	☐	☐	☐
b) Manager with employee responsibility	☐	☐	☐	☐	☐	☐
c) HR	☐	☐	☐	☐	☐	☐
d) Safety delegate / work environment representative	☐	☐	☐	☐	☐	☐
e) Union representatives	☐	☐	☐	☐	☐	☐
f) Occupational health service	☐	☐	☐	☐	☐	☐
g) Employees	☐	☐	☐	☐	☐	☐

14. To what extent are the following used as a <u>basis for decisions</u> regarding preventive/promotive work environment measures:

	Very low extent	Low extent	Neither low nor high extent	High extent	Very high extent	Do not know
a) Deficiencies in the work environment reported by employees	☐	☐	☐	☐	☐	☐
b) Analysis of employee data (e.g., sick leave, accidents, incidents)	☐	☐	☐	☐	☐	☐
c) Analysis of operational data (e.g., productivity, budget)	☐	☐	☐	☐	☐	☐
d) Analysis combining employee and operational data (so-called HR analysis)	☐	☐	☐	☐	☐	☐
e) Expected profitability	☐	☐	☐	☐	☐	☐
f) Dialogue with safety delegates or union representatives	☐	☐	☐	☐	☐	☐
g) Dialogue with occupational health service	☐	☐	☐	☐	☐	☐
h) Dialogue with internal resources (e.g., HR, work environment coordinator, etc.)	☐	☐	☐	☐	☐	☐
i) Internal improvement processes / policies and goal documents	☐	☐	☐	☐	☐	☐
j) External requirements (e.g., laws, regulations, industry requirements)	☐	☐	☐	☐	☐	☐

Choice of work environment measures

15. To what extent is <u>the content</u> of preventive/promotive work environment measures based on:

	Very low extent	Low extent	Neither low nor high extent	High extent	Very high extent	Do not know
a) Dialogue with occupational health service	☐	☐	☐	☐	☐	☐
b) Dialogue with internal expert resource (e.g., HR, work environment coordinator, etc.)	☐	☐	☐	☐	☐	☐
c) Dialogue with safety delegates or union representatives	☐	☐	☐	☐	☐	☐
d) Dialogue with employees	☐	☐	☐	☐	☐	☐
e) Scientific knowledge or proven experience (i.e., evidence-based methods)	☐	☐	☐	☐	☐	☐
f) Knowledge of measures conducted in other organizations	☐	☐	☐	☐	☐	☐
g) The organization's conditions and situation	☐	☐	☐	☐	☐	☐
h) Existing work methods and tools	☐	☐	☐	☐	☐	☐
i) Previous experiences from my organization	☐	☐	☐	☐	☐	☐

16. The content of preventive/promotive work environment measures is usually <u>anchored</u> with:

	Strongly disagree	Some-what disagree	Neither agree nor disagree	Some-what agree	Strongly agree	Do not know
a) Managers at several levels	☐	☐	☐	☐	☐	☐
b) Manager with employee responsibility	☐	☐	☐	☐	☐	☐
c) Internal support functions (e.g., HR, work environment coordinator, etc.)	☐	☐	☐	☐	☐	☐
d) Employees	☐	☐	☐	☐	☐	☐
e) Union representatives	☐	☐	☐	☐	☐	☐
f) Occupational health service	☐	☐	☐	☐	☐	☐
g) Safety delegates	☐	☐	☐	☐	☐	☐

17. In my organization, I consider us to have:

a) Sufficient resources, such as time and personnel, to analyze underlying causes of challenges in the work environment

- ☐ Strongly disagree
- ☐ Somewhat disagree
- ☐ Neither agree nor disagree
- ☐ Somewhat agree
- ☐ Strongly agree
- ☐ Do not know

b) Sufficient knowledge to select relevant measures in the preventive/promotive work environment management

- ☐ Strongly disagree
- ☐ Somewhat disagree
- ☐ Neither agree nor disagree
- ☐ Somewhat agree
- ☐ Strongly agree
- ☐ Do not know

c) Access to appropriate methods and routines that can be used to select relevant work environment measures

- ☐ Strongly disagree
- ☐ Somewhat disagree
- ☐ Neither agree nor disagree
- ☐ Somewhat agree
- ☐ Strongly agree
- ☐ Do not know

Evaluation of implemented work environment measures

18. To what extent is a plan made for how the results of work environment measures will be evaluated in connection with the decision on the measure?

Very low extent	Low extent	Neither low nor high extent	High extent	Very high extent	Do not know
☐	☐	☐	☐	☐	☐

19. To what extent are the results from previous work environment measures used to develop a long-term strategy in the ongoing work environment management?

Very low extent	Low extent	Neither low nor high extent	High extent	Very high extent	Do not know
☐	☐	☐	☐	☐	☐

Investments and costs

20. Which option best corresponds to how the cost of work environment measures is budgeted?

☐ The cost of ordering preventive/promotive work environment measures is charged to the respective manager's budget (manager with employee responsibility)

☐ The cost of work environment measures is centralized in the organization (does not affect the budget of the manager with employee responsibility)

21. To what extent do you believe that the <u>leadership considers</u> it important to invest in:

	Very low extent	Low extent	Neither low nor high extent	High extent	Very high extent	Do not know
a) Improved work environment	☐	☐	☐	☐	☐	☐
b) Competitive salaries	☐	☐	☐	☐	☐	☐
c) Competence development	☐	☐	☐	☐	☐	☐
d) Employer brand	☐	☐	☐	☐	☐	☐
e) Environmental sustainability	☐	☐	☐	☐	☐	☐

22. To what extent do you believe that your budget <u>allows</u> for investment in:

	Very low extent	Low extent	Neither low nor high extent	High extent	Very high extent	Do not know
a) Improved work environment	☐	☐	☐	☐	☐	☐
b) Competitive salaries	☐	☐	☐	☐	☐	☐
c) Competence development	☐	☐	☐	☐	☐	☐
d) Employer brand	☐	☐	☐	☐	☐	☐
e) Environmental sustainability	☐	☐	☐	☐	☐	☐

23. When you are about to implement preventive/promotive work environment measures, to what extent do you estimate <u>the total cost</u>?

Very low extent	Low extent	Neither low nor high extent	High extent	Very high extent	Do not know
☐	☐	☐	☐	☐	☐

24. Describe the <u>costs included</u> when estimating the total cost.

____ (Free text, 400 characters)

25. In connection with the implementation of preventive/promotive work environment measures, to what extent do you consider:

	Very low extent	Low extent	Neither low nor high extent	High extent	Very high extent	Do not know
a) The return on investment	☐	☐	☐	☐	☐	☐
b) Cost savings of the measures	☐	☐	☐	☐	☐	☐
c) Cost-effectiveness of the measure	☐	☐	☐	☐	☐	☐
d) Improvement of productivity	☐	☐	☐	☐	☐	☐
e) Improvement of work environment	☐	☐	☐	☐	☐	☐
f) Improvement of employee health	☐	☐	☐	☐	☐	☐

26. Compared to the current situation, to what extent would you like the following to be considered more when implementing preventive/promotive work environment measures:

	Much less	Less	Neither less nor more	More	Much more	Do not know
a) The return on investment	☐	☐	☐	☐	☐	☐
b) Cost savings of the measure	☐	☐	☐	☐	☐	☐
c) Cost-effectiveness of the measure	☐	☐	☐	☐	☐	☐
d) Improvement of productivity	☐	☐	☐	☐	☐	☐
e) Improvement of work environment	☐	☐	☐	☐	☐	☐
f) Improvement of employee health	☐	☐	☐	☐	☐	☐

27. What would be needed or required to consider, estimate, or calculate the answer options in the previous question to a greater extent?

____ (Free text, 400 characters)

Occupational health service

28. Which option best corresponds to the type of occupational health service you have? (Question 29-32 is NOT answered if answering "do not have access" here)

- ☐ Internal occupational health service
- ☐ External occupational health service
- ☐ We do not have access to occupational health service

29. Which option best corresponds to the agreement you have with the occupational health service?

- ☐ Subscription agreement with full-service package: an annual fee (charged to the organization or centrally) where all services are available
- ☐ Subscription agreement with basic services and ordering: an annual fee where some basic services are included, while other services are ordered separately
- ☐ Ordering agreement with basic fee: an annual fee, but without any specified services included in the amount, all services are purchased as needed
- ☐ Ordering agreement without basic fee: there is no basic fee, all services are purchased as needed
- ☐ Do not know

30. Which option best corresponds to how the ordering of preventive/promotive work environment measures is <u>usually done</u> with the occupational health service?

- ☐ Ordering of preventive/promotive work environment measures is based on pre-packaged measures
- ☐ Ordering of preventive/promotive work environment measures is based on pre-packaged measures, somewhat tailored to our needs
- ☐ Ordering of preventive/promotive work environment measures is always tailored based on dialogue between the requester and the occupational health service
- ☐ We do not order preventive/promotive work environment measures from the occupational health service
- ☐ Do not know

31. Which option best corresponds to your collaboration with the occupational health service?

- ☐ We meet as needed in connection with individual assignments
- ☐ We have regular meetings in addition to individual assignments to share information and follow up on the work environment management
- ☐ We have close collaboration where the occupational health service carries out individual assignments, participates in coordination meetings, and is a natural part of our work environment management
- ☐ Do not know

32. Overall, we have good collaboration with the occupational health service in the preventive/promotive work environment management:

Strongly disagree	Somewhat disagree	Neither agree nor disagree	Somewhat agree	Strongly agree
☐	☐	☐	☐	☐

Trade union collaboration

33. Do you have collective agreements?

☐ Yes

☐ No

34. To what extent do you have a well-functioning cooperation agreement where the employer and union representatives participate?

Very low extent	Low extent	Neither low nor high extent	High extent	Very high extent	No cooperation agreement
☐	☐	☐	☐	☐	☐

Overall picture

35. According to you, what are the most common challenges in your work environment?

- ☐ Imbalance between demands and resources
- ☐ Threats and violence
- ☐ Harassment and bullying
- ☐ Conflicts
- ☐ Physical strain
- ☐ Noise, air, and chemical exposure
- ☐ Accidents or safety risks
- ☐ Other (free text)

36. To what extent do you believe that your systematic work environment management prevent work-related health problems and/or accidents?

Very low extent	Low extent	Neither low nor high extent	High extent	Very high extent
☐	☐	☐	☐	☐

37. To what extent do you believe that your systematic work environment management promote health?

Very low extent	Low extent	Neither low nor high extent	High extent	Very high extent
☐	☐	☐	☐	☐

38. What do you think would make your preventive/promotive work environment management more successful?

______________________________ (Free text, 400 characters)

Organization

The final questions concern your organization.

39. Approximately how many employees are in the organization referred to in the survey?

Enter the number in digits: _______________

40. Which sector does your organization belong to?

- ☐ Private
- ☐ Municipality
- ☐ Region
- ☐ State

41. Which industry best describes your organization?

- ☐ Trade
- ☐ Hotel and restaurant operations
- ☐ Culture, entertainment, and leisure
- ☐ Labor-intensive service (rental, property services, support services, and other services)
- ☐ Knowledge-intensive service (law, science, technology, finance, and real estate operations)
- ☐ Information and communication activities
- ☐ Public administration and defense
- ☐ Education
- ☐ Health and care; social services
- ☐ Construction
- ☐ Agriculture, forestry, and fishing
- ☐ Transport and storage
- ☐ Manufacturing, production of goods and energy

42. In which county is your organization located?

- ☐ Blekinge
- ☐ Dalarna
- ☐ Gotland
- ☐ Gävleborg
- ☐ Halland
- ☐ Jämtland
- ☐ Jönköping
- ☐ Kalmar
- ☐ Kronoberg
- ☐ Norrbotten
- ☐ Skåne
- ☐ Stockholm
- ☐ Sörmland
- ☐ Uppsala
- ☐ Värmland
- ☐ Västerbotten
- ☐ Västernorrland
- ☐ Västmanland
- ☐ Västra Götaland
- ☐ Örebro
- ☐ Östergötland
