# Supplementary material for: Examining preventive occupational health and safety management in the Swedish welfare sector–questionnaire development, its validity and reliability, and initial findings on employers’ knowledge
Source: PLoS One. 2024 Nov 14;19(11):e0311788. doi: 10.1371/journal.pone.0311788 (PMC11563452; doi:10.1371/journal.pone.0311788)
Supplement: S3 File — (PDF) [file pone.0311788.s003.pdf]

S3 Examples of the inductive qualitative analysis process

Theme	Sub-theme	Category	Sub-category	Code	Quote
Organisational-level measures within systematic work environment management	Workplace interventions	Structural measures	Operations design	Distribution of work tasks	"Created new work positions to divide work tasks among a larger number of employees, with varying levels of responsibility."
