# Supplementary material for: Examining preventive occupational health and safety management in the Swedish welfare sector–questionnaire development, its validity and reliability, and initial findings on employers’ knowledge
Source: PLoS One. 2024 Nov 14;19(11):e0311788. doi: 10.1371/journal.pone.0311788 (PMC11563452; doi:10.1371/journal.pone.0311788)
Supplement: S4 File — (PDF) [file pone.0311788.s004.pdf]

ID	Sector	sufficient knowledge on preventive OHSM
1	1	3
2	1	3
6	1	4
7	3	4
8	1	4
10	1	4
11	1	5
12	1	3
13	3	4
14	2	4
16	2	4
18	2	4
20	2	4
21	2	4
22	2	3
23	2	5
24	2	4
25	3	5
26	2	4
27	2	5
28	2	4
29	2	5
30	2	4
31	2	2
32	2	4
33	2	2
34	2	4
35	2	3
36	3	4
39	2	3
40	2	3
43	2	3
44	2	4
45	2	3
46	3	4
48	2	4
49	2	4
50	2	4
52	2	4
53	1	3
55	2	4
56	2	5
59	2	5
60	2	4
61	2	4
62	2	3
63	2	4
65	3	4
70	2	4

71	2	4
72	2	3
73	2	5
75	2	3
77	2	2
79	3	4
80	2	5
81	2	3
82	3	5
83	2	4
84	2	5
89	2	2
90	2	4
96	3	4
98	1	2
99	2	4
101	2	4
102	2	4
103	1	3
104	2	4
105	2	4
107	2	4
108	2	4
109	2	2
110	2	3
112	2	4
113	2	5
114	2	4
118	2	4
119	2	4
123	1	5
125	2	4
126	2	3
127	2	4
128	1	5
129	1	4
130	1	4
133	2	3
134	1	4
135	2	5
136	1	5
137	1	5
139	1	4
141	1	1
142	1	4
143	3	4
144	3	2
145	2	3
146	1	4
148	2	5

151	3	4
152	2	4
153	1	5
154	2	4
155	1	4
156	3	5
159	2	4
160	3	4
161	3	2
164	2	5
166	3	4
169	3	4
170	2	2
174	3	4
176	2	4
177	2	3
179	3	4
182	3	4
184	3	3
185	1	4
186	1	5
189	3	4
191	3	.

Sector: 1=Private, 2=Municipality, 3=Region

Sufficient knowledge: 1=Strongly disagree, 2=Somewhat disagree, 3=Neither agree nor disagree, 4=Somewhat agree, 5=Strongly agree
